# Case Report: Portable hypothermic machine perfusion for interregional deceased-donor kidney sharing in Vietnam

**DOI:** 10.3389/frtra.2026.1982759

**Published:** 2026-10-21

**Authors:** Hiep Nhu Pham, Xuan Thanh Nguyen, Khanh Ngoc Tran, Dung Duc Nguyen, Anh Van Quoc Nguyen, Kha Cong Le Bui, Hung Ngoc Pham

**Affiliations:** 1 Department of Pediatric Surgery and Abdominal Emergency, Hue Central Hospital, Hue, Vietnam; 2 Department of Urology B, Hue Central Hospital, Hue, Vietnam; 3 Department of Thoracic and Cardiovascular Surgery, Hue Central Hospital, Hue, Vietnam; 4 Department of Urology A, Hue Central Hospital, Hue, Vietnam

**Keywords:** case report, deceased donor, hypothermic machine perfusion, interregional kidney sharing, kidney transplantation, organ preservation

## Abstract

Long-distance transportation can prolong cold ischemia and complicate the utilization of deceased-donor kidneys, particularly in geographically dispersed transplant systems. We report, to our knowledge, the first clinical implementation in Vietnam of portable hypothermic machine perfusion (HMP) during interregional kidney transport. A kidney from a 61-year-old expanded-criteria deceased donor was allocated to a 42-year-old man with end-stage renal disease who had been receiving hemodialysis for 2 years following failure of a previous kidney allograft. The graft was transported over 1,000 km by ground transfer and scheduled commercial flight and was preserved using a LifePort Kidney Transporter with KPS-1 solution under pressure-controlled HMP at 30 mmHg. Total cold ischemia time was 10 h 50 min. From HMP initiation to arrival, renal vascular resistance decreased from 1.68 to 0.44 mmHg/mL/min, while perfusate flow increased from 16 to 52 mL/min. Despite three separate renal arteries and a second warm ischemia time of 55 min, the graft produced urine immediately after reperfusion. The recipient required no dialysis during the first postoperative week and maintained stable graft function at 6 months, with a serum creatinine of 142 µmol/L and an estimated glomerular filtration rate of approximately 55 mL/min/1.73 m^2^. This case demonstrates the operational feasibility of portable HMP for long-distance, interregional deceased-donor kidney sharing in Vietnam.

## 1 Introduction

Kidney transplantation offers superior overall survival and quality of life compared with maintenance dialysis for patients with end-stage renal disease (ESRD) (1, 2). In Vietnam, transplantation has expanded since the first kidney transplant in 1992 and the introduction of deceased-donor transplantation in 2010. However, organ availability remains limited, while the number of patients with chronic kidney disease (CKD) continues to rise, leading to the increasing use of less optimal grafts including organs from expanded criteria donors (ECD) (3).

Static cold storage (SCS) remains the standard method of kidney preservation because of its simplicity and widespread availability. However, it provides neither active perfusion nor real-time flow and resistance data, limitations that are particularly relevant during long-distance transport. In Vietnam, interregional organ sharing often depends on scheduled commercial flights, which may prolong cold ischemia time (CIT) and increase ischemia–reperfusion injury (IRI) and the risk of delayed graft function (DGF) (4).

Hypothermic machine perfusion (HMP) provides continuous cold perfusion and permits serial assessment of perfusion parameters during transport. Compared with SCS, HMP has been associated with a lower incidence of DGF, particularly in kidneys from higher-risk donors (5).

To our knowledge, this is the first reported clinical implementation of portable HMP in Vietnam for an expanded-criteria donor kidney transported by commercial flight over more than 1,000 km. This report focuses on the practical integration of HMP into a long-distance organ-sharing pathway and the recipient's early outcome.

## 2 Case description

A 61-year-old deceased donor with hypertension, type 2 diabetes mellitus, and ischemic heart disease was declared brain dead after severe polytrauma with a Glasgow Coma Scale score of 3 (6). The donor met the conventional ECD definition by age alone (≥60 years) (7) The donor met the conventional ECD definition by age alone (≥60 years) (7) and was anti-HCV antibody positive; donor HCV RNA, detailed hemodynamic data, vasopressor requirements, terminal urine output, and ICU duration were unavailable. Serum creatinine transiently increased to 229 µmol/L and subsequently decreased to 82 µmol/L before procurement. Preimplantation biopsy was not routinely performed. The contralateral kidney was transplanted elsewhere, but its preservation and recipient outcomes were unavailable.

The recipient was a 42-year-old man receiving hemodialysis for 2 years after failure of a kidney allograft implanted 11 years earlier. The original cause of ESRD and pretransplant residual urine output were not documented. The graft failed after progressive chronic dysfunction clinically attributed to chronic rejection, although no confirmatory biopsy report was available, and remained *in situ* in the right iliac fossa. Donor–recipient assessment demonstrated ABO compatibility and two of six HLA mismatches: none at HLA-A, one at HLA-B, and one at HLA-DR. Qualitative screening for anti-HLA class I and class II antibodies by ELISA was negative; a numerical cPRA value was not available. No preformed donor-specific anti-HLA antibodies were detected in the pretransplant serum using a Luminex single-antigen bead assay. An urgent pretransplant donor–recipient lymphocyte crossmatch performed by flow cytometry was negative. HLA typing data from the first donor were unavailable; therefore, repeated HLA mismatches could not be assessed. The recipient was anti-HCV antibody positive with detectable HCV RNA before DAA therapy; CMV IgG was positive and CMV IgM negative. A 12-week course of sofosbuvir/velpatasvir was planned according to the institutional protocol. The kidney was allocated to our center by the National Organ Transplant Coordination Center through the national deceased-donor sharing pathway, independently of this study. Allocation followed national principles of biological compatibility, equity, waiting-list priority, and transport feasibility.

After aortic cross-clamping, the kidney remained *in situ* without perfusion or cooling for 20 min (first WIT) before cold flushing with Custodiol. CIT, defined from initiation of cold flushing to removal from HMP, was 10 h 50 min. Pre-HMP SCS lasted 1 h 35 min, including approximately 30 min of back-table preparation. The intact aortic patch containing all three renal arterial ostia was secured within a single cannula, permitting perfusion of all three arteries. Nonoxygenated HMP was performed using 1,000 mL KPS-1 at 30 mmHg for an elapsed 9 h 15 min; active perfusion was approximately 8 h 45–55 min after excluding the ice-replenishment pause. No post-HMP SCS was used. Following procurement in Ho Chi Minh City, the kidney was transferred by road to the airport, transported on a single direct scheduled flight of approximately 1.5 h to Hue, and then transferred by road to the recipient center (Figure 1). Under the 2018 cooperation agreement between the National Organ Transplant Coordination Center and Vietnam Airlines, the LifePort was carried in the business-class cabin under the direct supervision of recipient-centre personnel trained in its operation. The device operated on battery power without requiring an external electrical connection. The team carried sterile supplies and additional ice and serially reviewed the device readings and alarms throughout transportation.

**Figure 1:**
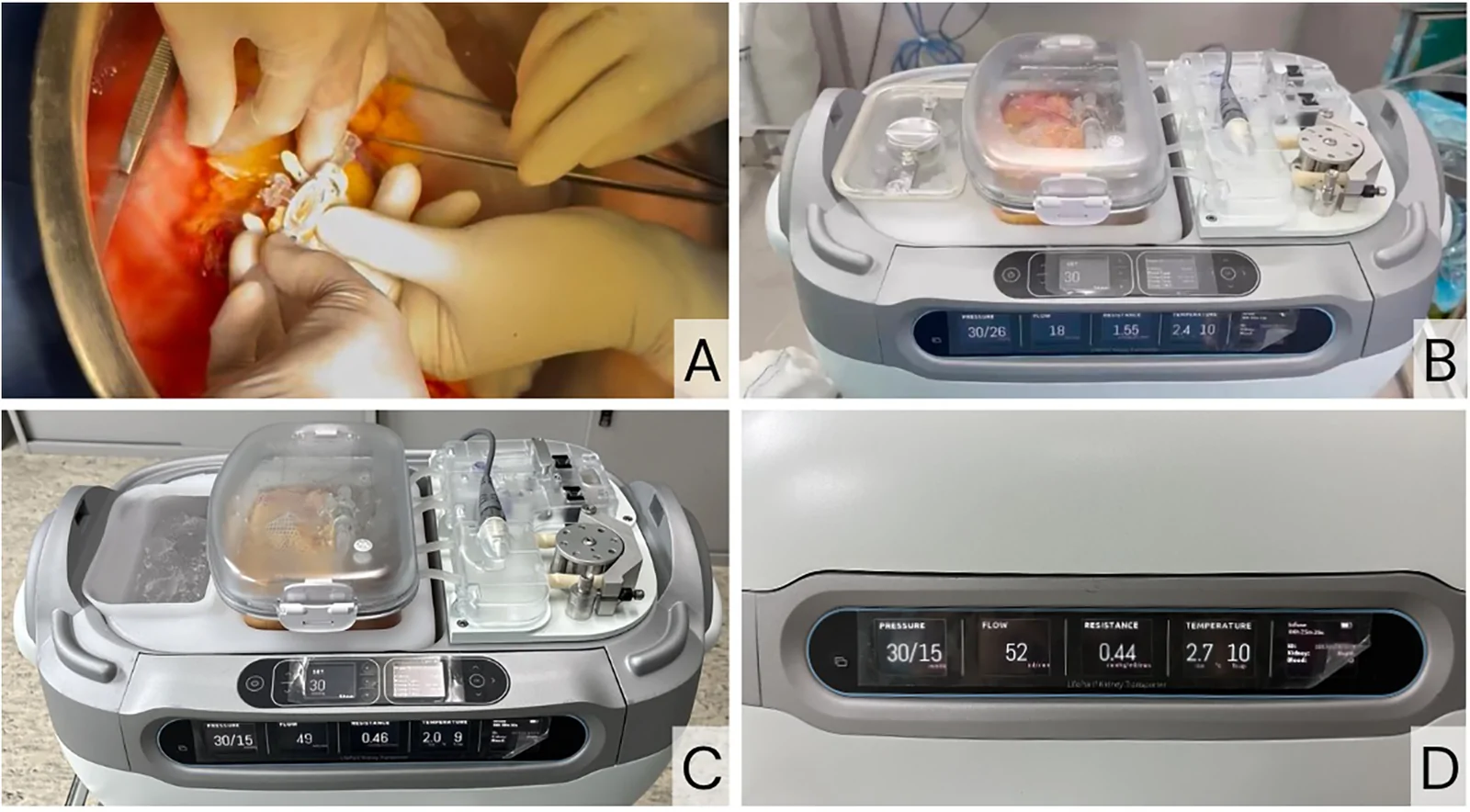
Hypothermic machine perfusion of the donor kidney during long-distance transport using the lifePort kidney transporter**. (A)** *ex vivo* cannulation of the intact donor aortic patch containing all three renal arterial ostia before HMP. **(B)** At 1 h, RVR was 1.55 mmHg/mL/min and flow was 18 mL/min. **(C)** At 9 h, RVR decreased to 0.46 mmHg/mL/min and flow increased to 49 mL/min. **(D)** On arrival at our hospital, RVR was 0.44 mmHg/mL/min and flow was 52 mL/min.

The HMP record showed a temporary interruption approximately 3.5–4 h after initiation. Perfusion was intentionally paused for approximately 20–30 min to replenish ice in the transporter. During this interval, device-recorded flow, pressure, and displayed resistance fell to zero, while the bubble-trap temperature increased transiently to approximately 15 °C. Perfusion subsequently resumed. At approximately 9 h 15 min, HMP was stopped as planned at the recipient center before the kidney was removed for implantation. The elapsed time displayed in the device record includes the temporary interruption and should not be interpreted as uninterrupted active perfusion time. At HMP initiation, RVR was 1.68 mmHg/mL/min and perfusate flow was 16 mL/min; at 1 h, the corresponding values were 1.55 mmHg/mL/min and 18 mL/min. By arrival, RVR had decreased to 0.44 mmHg/mL/min and flow had increased to 52 mL/min (Figure 2). The protocol target temperature range was 2–8 °C. During active HMP, ice-compartment readings ranged from approximately 1.5 °C to 7 °C, whereas bubble-trap readings were predominantly 9–10 °C and increased transiently to approximately 15 °C during the interruption. Renal parenchymal temperature was not measured.

**Figure 2:**
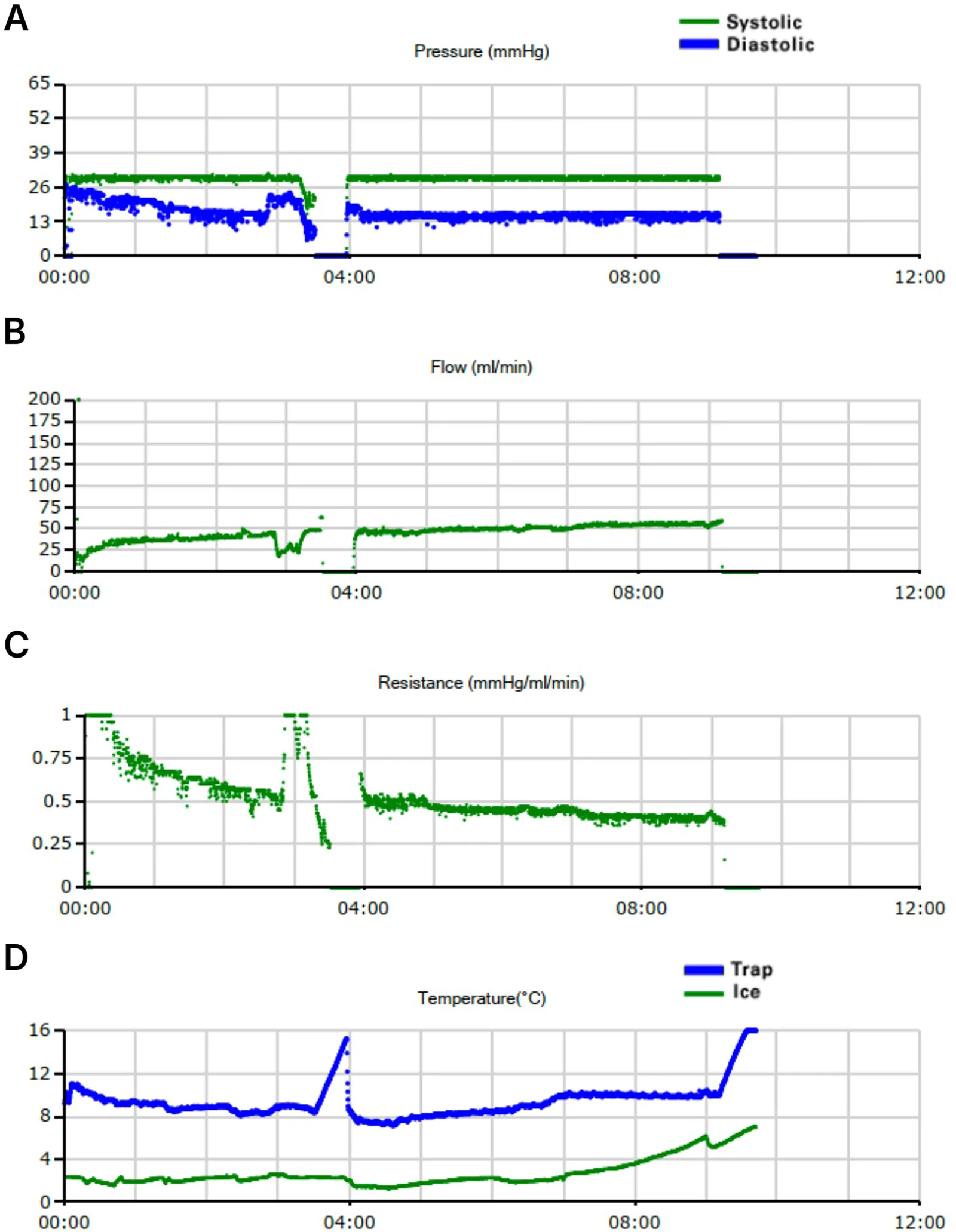
Perfusion and temperature profiles recorded by the lifePort kidney transporter during interregional transport. **(A)** Systolic (green) and diastolic (blue) perfusion pressures, expressed in mmHg. **(B)** Renal perfusate flow, expressed in mL/min. **(C)** Calculated renal vascular resistance, expressed in mmHg/mL/min. **(D)** Bubble-trap (blue) and ice-compartment (green) sensor temperatures, expressed in °C. The horizontal axis represents elapsed time since HMP initiation, including the temporary interruption. The zero-value interval at approximately 3.5–4 h corresponds to a 20-30-minute pause for ice replenishment, whereas the shorter terminal deflection near 9.5 h represents planned cessation of HMP at the recipient center before graft removal and implantation. The ice-compartment sensor Reading increased from approximately 1.5 °C to 7 °C during the final 3 h, whereas the bubble-trap sensor recorded temperatures of approximately 9-10 °C during substantial periods of active perfusion. These values are device sensor readings and do not represent direct measurements of kidney parenchymal temperature.

Because all three renal arterial ostia were contained within the cannulated aortic patch, the recorded flow and vascular resistance values represent aggregate perfusion through the entire cannulation system rather than perfusion through a single dominant artery. However, these device-derived measurements cannot determine the relative distribution of perfusate among the three individual renal arteries. The graft was subsequently implanted in the left iliac fossa. The renal vein was anastomosed to the external iliac vein, and the three renal arteries were separately anastomosed to the external iliac artery. The second WIT, defined as the interval from removal of the graft from cold preservation to reperfusion, was 55 min, reflecting the vascular complexity and the technical considerations associated with retransplantation. After reperfusion, the graft showed uniform cortical coloration and satisfactory turgor with immediate urine production (Figure 3).

**Figure 3:**
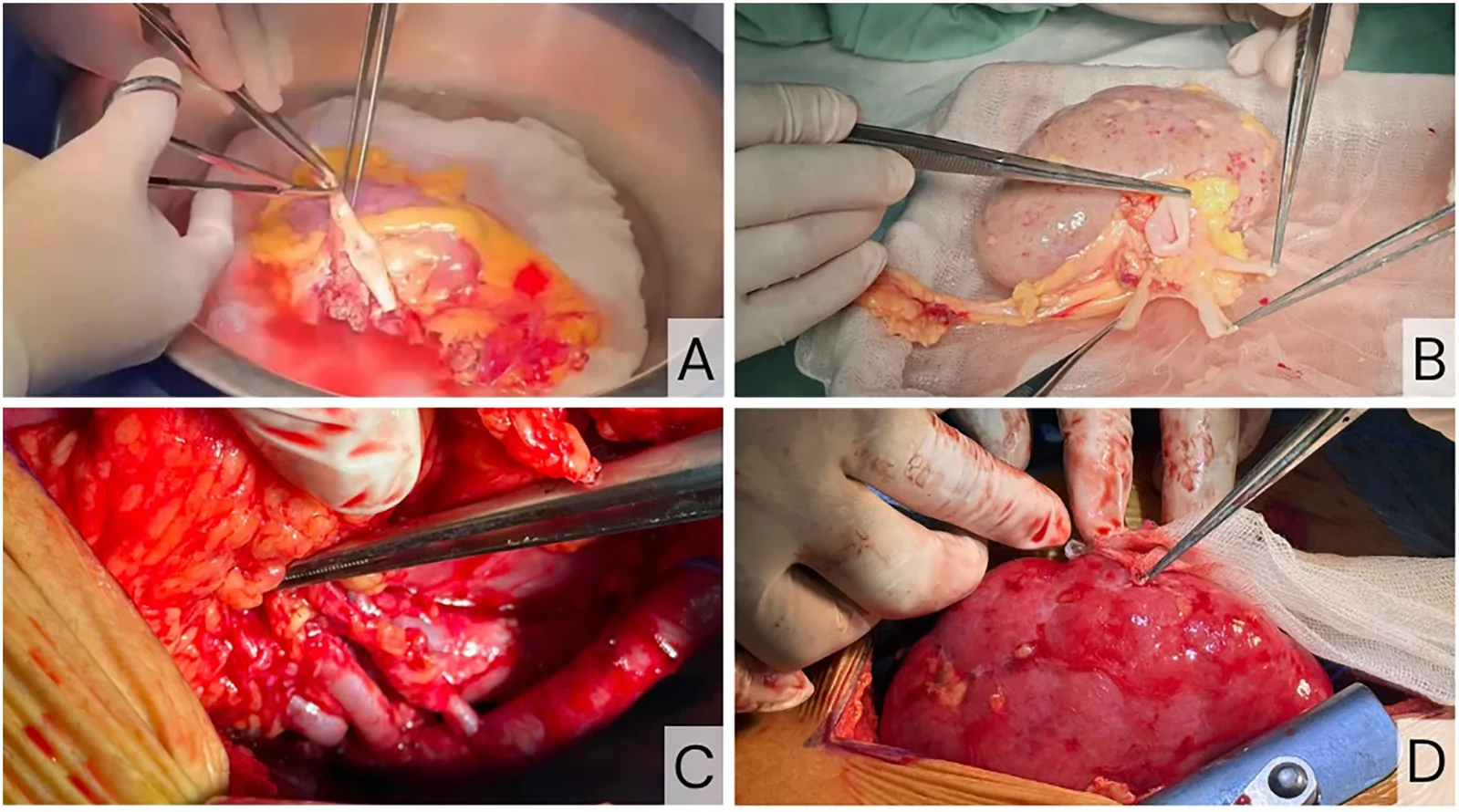
Preparation and implantation of the donor kidney allograft. **(A)** Inspection of the graft in cold preservation solution. **(B)** Back-table dissection of the renal hilum with identification of the renal artery, vein and ureter. **(C)** Completion of three separate arterial anastomoses. **(D)** Graft appearance immediately after reperfusion, showing uniform cortical perfusion.

Although previous transplantation was recognized as a potential sensitizing event, the available pretransplant evaluation showed no detectable class I or class II anti-HLA antibodies, no preformed DSA, a negative flow-cytometric crossmatch, and only two of six HLA mismatches. On the basis of this immunological profile, basiliximab was selected rather than lymphocyte-depleting induction. Induction immunosuppression consisted of basiliximab administered intravenously on days 0 and 4, together with methylprednisolone. Initial maintenance therapy included tacrolimus, mycophenolate mofetil, and prednisolone. DAA treatment was initiated during the peri-transplant period with Sofosbuvir and Velpatasvir. The early postoperative course was uncomplicated. Urine output reached 4,600 mL during the first 24 h and remained between 3,000 and 4,000 mL/day thereafter. Serum creatinine was 1,227 µmol/L before transplantation, 1,064.9 µmol/L on postoperative day 1, and 872.1 µmol/L on day 2, corresponding to a day-1-to-day-2 creatinine reduction ratio of 18.1%. It subsequently decreased to 664 µmol/L on day 5, 301 µmol/L on day 9, and 154 µmol/L on day 21. No dialysis was required during the first postoperative week; thus, the conventional dialysis-based definition of DGF was not met, although the creatinine trajectory was consistent with slow graft function. Doppler ultrasonography on postoperative day 10 showed an intrarenal resistive index of 0.66 with a peak systolic velocity of 35 cm/s; at the renal artery origin/anastomotic region, the corresponding values were 0.62 and 70 cm/s. The recipient was discharged on postoperative day 28 with stable graft function. After completing the planned 12-week sofosbuvir/velpatasvir course, the recipient was switched from tacrolimus to cyclosporine A because of upper- and lower-limb tremor and sleep disturbance attributed to tacrolimus. Cyclosporine A and sofosbuvir/velpatasvir were therefore not coadministered. Subsequent cyclosporine trough concentrations were 93–117 ng/mL. HCV RNA was undetectable following completion of antiviral treatment. At 6-month follow-up, serum creatinine was 142 µmol/L, corresponding to an eGFR of approximately 55 mL/min/1.73 m^2^ calculated using the 2021 CKD-EPI creatinine equation. Urinalysis was protein-negative at the available approximately 3- and 6-month visits. Rejection surveillance was clinical and serological: repeat Luminex single-antigen bead testing remained negative for donor-specific anti-HLA antibodies, and no clinically treated acute rejection occurred.

## 3 Discussion

The principal contribution of this case is logistical rather than evidence that HMP was clinically necessary. Transport over more than 1,000 km created geographic and organizational complexity; however, the CIT of 10 h 50 min was not unusually prolonged and does not establish that HMP was required. Transfer was enabled by advance coordination with the national allocation center and airline, cabin carriage, battery operation, trained escort personnel, and additional ice and sterile supplies. Programs adopting portable HMP should define responsibilities for serial device checks, ice replenishment, temperature excursions, transport delays, and handover at the recipient center. This case demonstrates operational feasibility, not superiority over SCS.

The donor kidney had several risk factors for early dysfunction. Despite immediate diuresis and no dialysis during the first postoperative week, the day-1-to-day-2 creatinine reduction ratio was 18.1%, and creatinine remained 664 µmol/L on day 5 before decreasing to 154 µmol/L on day 21, consistent with slow graft function rather than immediate functional recovery. Although HMP may reduce DGF in ECD kidneys (5), this outcome cannot be attributed to HMP alone. Potential pharmacokinetic interactions between direct-acting antivirals and calcineurin inhibitors should be considered in HCV-positive transplant recipients. Cyclosporine may increase systemic exposure to sofosbuvir and velpatasvir when these agents are coadministered. In this case, however, the planned 12-week course of sofosbuvir/velpatasvir was completed before cyclosporine A was introduced; therefore, no concurrent exposure occurred. Kidney and liver function, HCV RNA, and calcineurin inhibitor trough concentrations were monitored during clinical follow-up. HCV RNA was undetectable following completion of antiviral treatment, and subsequent cyclosporine trough concentrations were 93–117 ng/mL. This treatment sequence avoided the need to manage simultaneous exposure to cyclosporine A and sofosbuvir/velpatasvir.

During HMP, RVR decreased while perfusate flow increased, providing an objective record of perfusion throughout transportation. These trends were reassuring but should not be considered definitive evidence of graft viability. Flow and resistance have limited predictive accuracy when used alone, and the decision to transplant should remain based on the combined assessment of donor features, kidney function, ischemic exposure, graft appearance and recipient factors (8). Because all three renal arterial ostia were contained within the cannulated aortic patch, the recorded flow and vascular resistance values represent aggregate perfusion through the entire cannulation system rather than perfusion through a single dominant artery. However, these device-derived measurements cannot determine the relative distribution of perfusate among the three individual renal arteries.

Temperature control was the principal operational limitation. Bubble-trap readings exceeded 8 °C for substantial periods despite ice replenishment, supporting the need for predefined surveillance thresholds and replenishment plans. Because renal parenchymal temperature was not measured and no comparator was available, the clinical effect of these sensor excursions cannot be determined.

This report is limited by its single case, the absence of an SCS comparator and preimplantation biopsy, and the relatively short follow-up. A numerical cPRA value and HLA typing data from the first donor were unavailable, which limited complete retrospective characterization of the recipient's immunological risk and prevented assessment of repeated HLA mismatches. Therefore, this report demonstrates operational feasibility rather than comparative effectiveness. The exact timing of the last pretransplant dialysis session and BK virus surveillance results could not be verified from the available records. Further prospective data are needed to evaluate clinical outcomes, transport-related complications and cost-effectiveness.

## 4 Conclusion

Portable HMP was integrated into the interregional transport of an expanded-criteria donor kidney over more than 1,000 km. The device record documented a temporary interruption for ice replenishment. The graft produced urine immediately after reperfusion and required no dialysis during the first postoperative week, while the gradual decline in serum creatinine was consistent with slow graft function. Kidney function was stable at 6 months. This single case demonstrates the operational feasibility of portable HMP for interregional deceased-donor kidney sharing in Vietnam but cannot establish a comparative clinical benefit over SCS.

## Data Availability

The original contributions presented in the study are included in the article/Supplementary Material, further inquiries can be directed to the corresponding author.
